# Selective sweep and mammary transcriptomics identify breed-specific lactation drivers in East Friesian dairy sheep

**DOI:** 10.1093/jas/skaf339

**Published:** 2025-09-29

**Authors:** Danni Li, Xueyang Zhao, Yuhang Xiao, Ran Li, Yu Jiang, Lei Zhang, Yuxuan Song

**Affiliations:** College of Animal Science and Technology, Northwest A&F University, Yangling, Shaanxi 712100, P.R. China; College of Animal Science and Technology, Northwest A&F University, Yangling, Shaanxi 712100, P.R. China; College of Animal Science and Technology, Northwest A&F University, Yangling, Shaanxi 712100, P.R. China; College of Animal Science and Technology, Northwest A&F University, Yangling, Shaanxi 712100, P.R. China; College of Animal Science and Technology, Northwest A&F University, Yangling, Shaanxi 712100, P.R. China; College of Animal Science and Technology, Northwest A&F University, Yangling, Shaanxi 712100, P.R. China; College of Animal Science and Technology, Northwest A&F University, Yangling, Shaanxi 712100, P.R. China

**Keywords:** *COL3A1* gene, dairy sheep, lactation performance, selection signaling, transcriptome

## Abstract

East Friesian sheep (EFR) are one of the world’s highest-performing sheep breeds for milk production. Whole-genome resequencing data from 45 EFR sheep were combined with published whole-genome data from 35 additional sheep. Population genetic analysis revealed that EFR sheep are genetically distinct from other breeds, with evidence of ancestral gene flow from other sheep lineages. Genome-wide selective sweep identified strong selection signals on chromosome 2, including extended haplotypes overlapping with QTL associated with milk protein content. Notably, missense mutations in the *COL3A1* and *COL5A2* genes—linked to mammary gland development—were detected within these regions. Based on these findings, molecular markers for lactation performance were derived and applied to EFR sheep selection. To further investigate lactation-related genes, we performed transcriptome sequencing of mammary gland tissue from lactating and dry crossbred dairy sheep (EFR male × Hu female). Differential expression analysis identified 2,178 significantly differentially expressed genes (DEGs), including the *COL3A1* gene, which was significantly downregulated. Integrating genomic and transcriptomic data, we confirmed *COL3A1* as a candidate gene influencing milk production traits. Notably, the *COL3A1* locus (g.130226140G>A) showed a significant association with milk yield in 1,019 EFR×Hu crossbred sheep. The GG genotype exhibited the highest milk yield, significantly outperforming AG (Δ = 11 kg, *P *< 0.05). These findings provide novel insights into the genetic basis of milk production in dairy sheep and offer valuable markers for breeding programs aimed at enhancing lactation performance.

## Teaser

A missense mutation in *COL3A1* is linked to increased milk yield in sheep, offering a genetic marker for improved dairy sheep breeding.

## Introduction

Growing demand for high-quality dairy has increased global sheep milk consumption. Compared with goat and cow milk, sheep milk contains higher levels of dry matter, milk fat, lactose, and beneficial fatty acids, and its β-casein profile closely resembles human milk, providing enhanced digestibility and strong market potential (Liu et al., 2025). The East Friesian (EFR) dairy sheep breed, often referred to as the “Holstein of dairy sheep,” is renowned for its high production efficiency, characterized by large udders, distinct morphological traits, and exceptional milk yields, though individual production can vary substantially (300–800 kg per lactation cycle) (Li et al., 2022). However, due to their poor adaptability and susceptibility to pneumonia, EFR sheep are primarily used in China for crossbreeding with local Hu sheep to improve lactation performance and develop new dairy sheep lines (Li et al., 2025).

Recent advances in genomic analyses have significantly enhanced our understanding of sheep trait genetics. Selective sweep analyses across multiple species have identified functional genes associated with key ovine characteristics, including reproduction, milk production, wool quality, and meat traits (Zhang et al., 2021, 2023; Rajawat et al., 2022). Complementary transcriptomic studies using RNA sequencing have further elucidated candidate genes involved in growth, development, reproduction, disease resistance, and mammary gland function (Hao et al., 2019; Shi et al., 2022). Extensive research has identified multiple genes regulating lactation traits in dairy sheep, such as *CSN2* (Suárez-Vega et al., 2015), *CSN3* (Li et al., 2024), *SOCS2* (Oget et al., 2019), and *SLC29A4* (Li et al., 2023). While the genetic architecture of milk production is complex, several key genes have been well characterized. The casein gene cluster on chromosome 6 demonstrates significant pleiotropic effects, influencing both milk yield and composition (Noce et al., 2016). Critical milk protein genes, including α-lactalbumin (LALBA) and β-lactoglobulin (LGB), are located within lactation-associated genomic regions on chromosome III (Padilla et al., 2018). The growth hormone receptor (GHR) gene plays a conserved role across breeds, affecting milk production in both Serra da Estrela and Sarda sheep (Vacca et al., 2013; Wang et al., 2015). In Sarda sheep specifically, GHR polymorphisms show significant associations with milk fat and protein yield, as well as lactose and urea content (Dettori et al., 2018, 2019).

This study integrates genome-wide selective sweep analysis with mammary gland transcriptomics to explore molecular markers and candidate genes potentially associated with lactation traits in East Friesian dairy sheep. Such an approach may help uncover key genetic loci relevant to milk yield, thereby providing breed-specific markers for molecular-assisted breeding and offering new perspectives on the genetic basis of lactation performance in dairy sheep.

## Materials and Methods

### Sample collection and whole-genome resequencing

**Figure 1.**
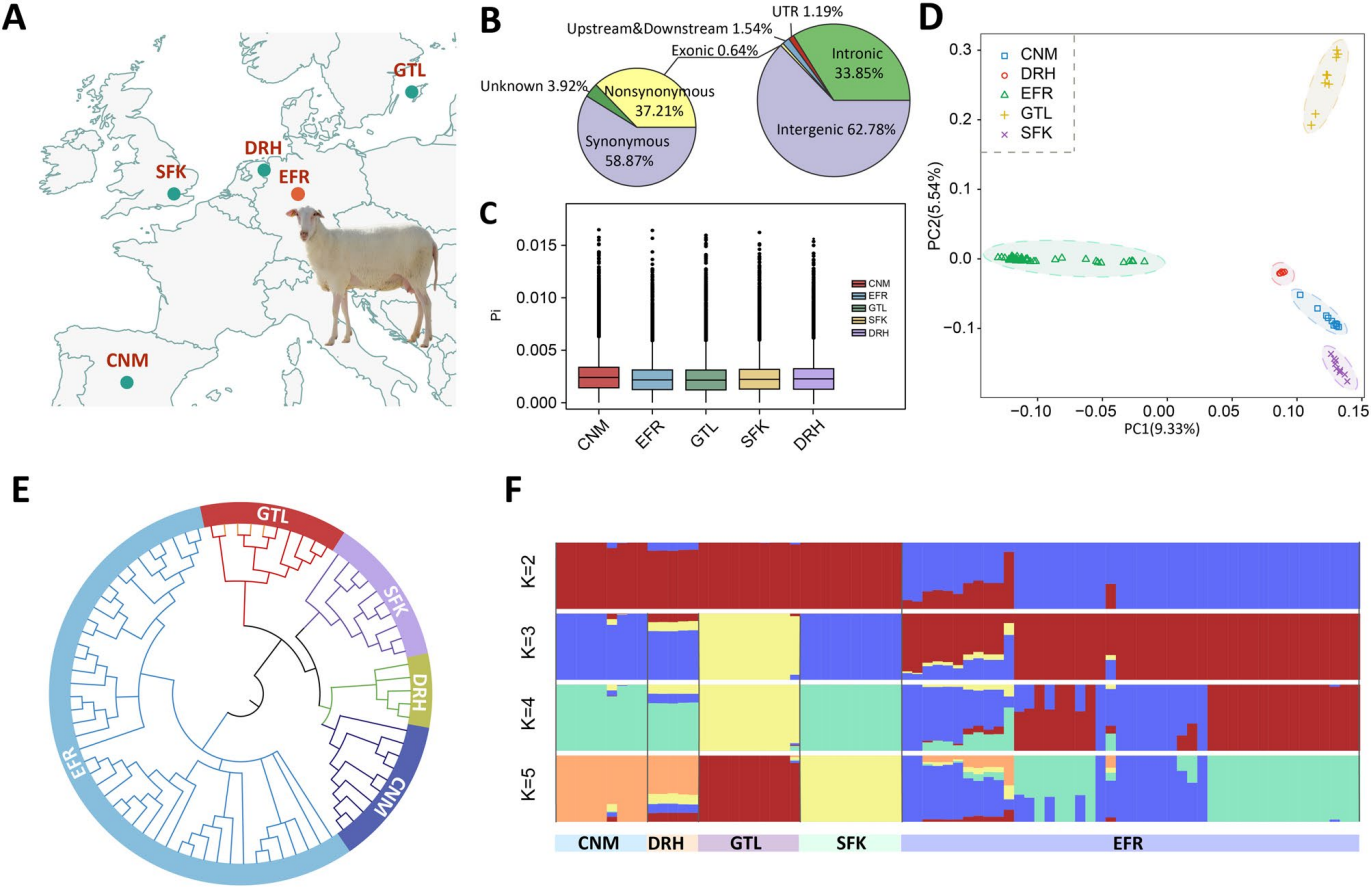
Geographic distribution and group structure of raw sheep populations in East Friesian. (A) Geographic locations of East Friesian and other sheep populations, indicated by red and green dots on a world map; (B) Functional classification of detected SNP; (C) Nucleotide diversity analysis; (D) Principal component analysis (PC1_vs_PC2); (E) Neighborhood dendrograms; (F) Population genetic component analysis.

A total of 35 EFR with multiple mating records were used in this study. These sheep were all obtained from the Hongguang Farm of Yuansheng Agriculture and Animal Husbandry Technology Co., Ltd (Gansu, China). Whole-genome resequencing of EFR sheep, with an average sequencing depth of 10.02×, was completed by Beijing Anuo Yoda Gene Technology Co., Ltd (Beijing, China). In addition, the whole-genome resequencing data of 10 EFR sheep with more than 20×, 5 Drenthe Heathen (DRH), 10 Gotland (GTL), 10 Suffolk (SFK), and 10 Chinese Merino sheep (CNM) in the Netherlands were downloaded from NCBI (https://www.ncbi.nlm.nih.gov/, BioProject: PRJNA624020). All comparator breeds were non-dairy varieties, contrasting with the dairy-optimized EFR (Fig. 1A and Table 1; Supplementary Table S1).

**Table 1.** Population of sheep selected in this study

Breeds	Abbreviations	Sample size	Phenotypic advantage
East Friesian	EFR	45	Milk production
Drenthe Heathen	DRH	5	Vegetation management, meat production
Suffolk	SFK	10	Meat production, wool production
Gotland	GTL	10	Short-tailed, pelt, and meat production
Chinese Merino	CNM	10	Wool production, meat production

### Data quality control and variation detection

Raw FASTQ files from whole-genome resequencing of EFR sheep were processed using Fastp v0.12.3 (Chen et al., 2018) with the following parameters: -q 20 -u 20 -n 5 -l 80 -w 8. Publicly available sequencing data from NCBI were converted from SRA to FASTQ format using Fastq-dump (v2.8.1;—gzip—split-3) and subjected to identical quality control. Clean reads were aligned to the Oar_rambouillet_v1.0 reference genome using BWA-MEM v0.7.13-r1126 (Li and Durbin, 2009) with default parameters. PCR duplicates were removed using Picard v.3.0.0 (https://broadinstitute.github.io/picard/, REMOVE_DUPLICATES=true). SNPs were initially called using GATK v4.0 (Nekrutenko and Taylor, 2012) and subsequently filtered to ensure data quality. Variants with a read quality score below 30, low quality by depth (QD < 2.0), strand bias (SOR > 3.0, FS > 60), low mapping quality (MQ < 40), or biased mapping and read position (MQRankSum < –12.5, ReadPosRankSum < –8.0) were excluded from further analysis (Wang et al., 2024). Further filtering was performed with BCFtools v1.13 (Danecek et al., 2021) to retain high-confidence SNPs by excluding variants with multiple alternative alleles, removing sites with more than 10% missing genotypes, and retaining only SNPs with a minor allele count greater than two. Finally, all SNPs were annotated using ANNOVAR (Wang et al., 2010).

### Population genetic analysis

Genetic diversity was assessed using VCFtools v0.1.16 (Danecek et al., 2011) by estimating nucleotide diversity (π) with a 50 kb window and 20 kb step size. This setting, commonly applied in livestock genomic studies, was chosen to balance resolution and statistical power, ensuring reliable detection of diversity patterns while minimizing noise (Song et al., 2024). For principal component analysis, the VCF files were first processed using EIGENSOFT (Abraham and Inouye, 2014). Smartpca v13050 was subsequently used to estimate the genetic relationships of the five populations. For population genetic component analysis, VCF files were first processed using PLINK v1.90b3.40 (Purcell et al., 2007), and ADMIXTURE v1.3.0 (Alexander and Lange, 2011) was subsequently used to analyze the genetic structure of the populations of five breeds totaling 80 sheep. For phylogenetic analyses, we used PLINK to calculate the distance matrix between pairs of individuals, constructed an NJ tree using MEGA v10.0 (Kumar et al., 2018), and visualized it using iTOL (Letunic and Bork, 2019).

### Genome-wide selective sweep

Fixation Index (Fst) was calculated using VCFtools v0.1.16 (Danecek et al., 2011) with the “—weir-fst-pop” option, which implements Weir and Cockerham’s unbiased estimator of Fst (Dar et al., 2020). A weighted average across SNPs was obtained within each 50 kb sliding window with a step size of 20 kb for the EFR and other sheep populations. Windows in the top 1% of the weighted Fst and log_e_(π_Other_/π_EFR_) values were identified as potentially under selection. The integrated haplotype score (iHS) was calculated using selscan v1.3.0 (Szpiech and Hernandez, 2014), which estimates extended haplotype homozygosity decay for ancestral and derived alleles, integrates the decay curves, and takes their log ratio. To control for allele frequency effects (An et al., 2024), raw iHS values were further normalized within frequency bins using norm v1.1.0 (Szpiech and Hernandez, 2014), yielding standardized iHS values. Windows in the top 1% of the corrected iHS values were filtered as potentially under selection. The intervals of the candidate-selected regions for each of the above three methods were annotated by ANNOVAR v2016-02-01 (Wang et al., 2010). The candidate regions were then characterized for long haplotypes and tagging SNPs using Haploview (Barrett et al., 2005).

### RNA-Seq and differential expression analysis

We performed transcriptome sequencing of mammary gland tissue from both lactating and non-lactating (dry) EFR (♂) × Hu sheep (♀) F2 crossbred individuals (*n* = 6 per physiological state). All animals were in their second parity, with lactating ewes sampled at mid-lactation (approximately 60–80 days in milk) and dry ewes sampled at 30 days after cessation of milking. The RNA libraries were sequenced on the Illumina NovaseqTM 6000 platform by LC Bio Technology Co., Ltd (Hangzhou, China). The transcriptome data were aligned to the sheep reference genome (Oar_rambouillet_v1.0) using HISAT2 v2-2.2.1 (Kim et al., 2015). The mapped reads for each sample were assembled using StringTie v2.1.6 (Pertea et al., 2015; Kovaka et al., 2019). We then estimated transcript expression levels using Ballgown and calculated FPKM values for mRNA expression level analysis. Differentially expressed gene (DEGs) analysis was performed between the two groups (lactation and dry milk stage) using DESeq2 (Love et al., 2014). DEGs were identified using the criteria |log_2_FC| > 1.5 and *P *< 0.05, with the dry milk stage (G) serving as the control group. Subsequently, we used KOBAS (Bu et al., 2021) (http://bioinfo.org/kobas/) to perform enrichment analysis of DEGs for GO function (Ashburner et al., 2000) and KEGG pathway (Kanehisa et al., 2021).

### Association analysis between target mutation with milk yield

We selected 1,019 F2 generation sheep for association analysis between milk yield and the target mutation site (*COL3A1* gene g.130226140G>A), and genotyping was performed using the Kompetitive Allele Specific PCR (KASP) technique (Dipta et al., 2024). The KASP genotyping was completed by Personal Biotechnology Co., Ltd (Shanghai, China). The specific primer information is as follows: Primer 1: GAAGGTGACCAAGTTCATGCTCTCCTTTAATTTCAGGGTGCTGC; Primer 2: GAAGGTCGGAGTCAACGGATTCTCCTTTAATTTCAGGGTGCTGT; Primer Common: CCTCTTTCTCCAGGCATTCCTT. The basic reaction system for KASP (5 μL) includes: 2.00 μL of DNA template, 2.50 μL of 2×KASP Master Mix, 0.004 μL of Mg^2+^, 0.056 μL of primer mixture, and 0.44 μL of ddH_2_O. The KASP amplification was performed on an LGC HydroCycler2 water bath PCR instrument. After the reaction was completed, genotyping was carried out using the high-sensitivity plate reader of LGC PheraStar.

Excel functions were employed to calculate population genetic parameters, including gene frequency, genotype frequency, purity, and heterozygosity. Correlation analysis with genotypic data was conducted using IBM SPSS Statistics 23.0 (IBMCorp., Armonk, NY, USA). A general linear model was applied, with milk yield as the dependent variable and genotype as the fixed effect. Parity and age of the ewes were included as covariates to account for potential confounding effects.

## Results

### Genetic analysis of raw sheep populations in EFR

This study analyzed whole-genome sequences from 45 EFR sheep and 35 comparator European breeds (80 total samples), identifying 30,640,186 high-confidence SNPs. The SNPs were predominantly distributed in intergenic (62.78%) and intronic (33.85%) regions, with smaller proportions in coding (0.64%; 37.21% non-synonymous, 58.87% synonymous), regulatory (1.29%), and untranslated regions (1.04%) (Fig. 1B). Nucleotide diversity analysis revealed that EFR sheep showed slightly lower genetic variation compared with the other evaluated breeds (Fig. 1C), with a pattern resembling that of Gotland sheep. Although the differences among breeds were small, this trend may reflect the influence of artificial selection in specialized dairy breeds.

Population structure analyses (PCA, ADMIXTURE, and Neighbor-Joining Tree) revealed genetic relationships between EFR sheep and non-dairy breeds. The first 10 principal components accounted for 40.21% of total genetic variance, with PC1 alone explaining 10.90% and clearly distinguishing EFR from other breeds (Fig. 1D). NJ tree and ADMIXTURE analyses corroborated these findings (Fig. 1E and F). At *K* = 2, EFR formed a distinct cluster while showing minor ancestral components from other breeds, consistent with their historical origin in northern Germany and the Netherlands. When *K* = 3, Gotland sheep are separated from Suffolk sheep. At higher *K*-values (4-5), EFR individuals subdivided into two subgroups, suggesting the presence of three distinct family lines within this population. Notably, Drenthe Heath sheep showed remarkable genetic similarity to Chinese Merino sheep, aligning with their documented breeding history.

### Genome-wide selective sweep in EFR sheep

**Figure 2.**
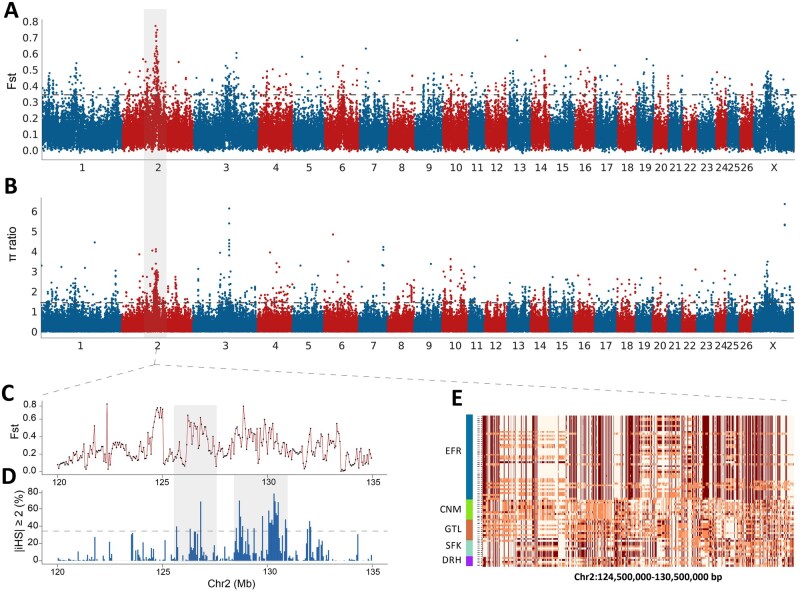
Characterization of selective sweep in East Friesian and other breeds. (A) Fst distribution, computed using a 50 kb sliding window of 20 kb steps, with the dashed line representing the top 1% threshold; (B) π ratio value distribution, computed using a 50 kb sliding window of 20 kb steps, with the dashed line representing the top 1% threshold; (C) Pairwise Fst values around candidate regions without overlap within a 50 kb window of candidate regions to calculate pairwise Fst values around candidate regions in 20 kb steps for a 50 kb window; (D) Proportion of SNPs with |iHS| ≥ 2 in a 50 kb window where candidate regions do not overlap; (E) Haplotype analysis of the strongest selected region in East Friesian sheep.

Population genetic analyses were performed to identify signatures of selection in EFR. We calculated FST to identify genomic regions with significant genetic differentiation between EFR and comparator breeds. Using the top 1% of weighted FST values as the selection threshold (Fig. 2A), we identified 561 candidate selective sweep regions containing 641 annotated genes (Supplementary Table S2). Notably, the strongest selection signals clustered within the 124–133 Mb interval on chromosome 2, suggesting this region may harbor genes crucial for EFR-specific traits.

Nucleotide diversity (π) comparisons between EFR and non-dairy breeds revealed 1,401 candidate selective sweep regions (top 1% of log_e_(π_Other_/π_EFR_) containing 1,066 annotated genes (Fig. 2B, Supplementary Table S3). Notably, the *KITLG* gene was identified within a significant 134.28–134.33 Mb region on chromosome 3.

To specifically assess EFR selection signals, iHS analysis was conducted independently. Using 50 kb sliding windows with a top 1% threshold, we identified 494 candidate regions harboring 558 genes, with the most significant signal at the *LIN28B* (chr8:35.60–35.65 Mb; Supplementary Table S4).

Intersection of FST, π ratio, and iHS results yielded 26 consensus genes (Table 2) with 19 shared selective sweep windows, predominantly clustered within chr2:126–130 Mb (Fig. 2C and D). This region contained an extended 6 Mb haplotype in EFR sheep (Fig. 2E) that completely overlapped with known quantitative trait loci (QTL) for milk protein percentage (Supplementary Table S5), strongly suggesting its functional importance for lactation performance.

**Table 2.** Table of candidate regions screened based on Fst, π ratio, and iHS

Chr	Region (Mb)	Max Fst	**|iHS|** ≥ **2 (%)**	**Max** π **ratio**	Candidate gene
1	126.42–126.45	0.3788	52.04	1.4556	*LOC101109936, POGK*
2	90.24–90.25	0.3843	46.75	1.4392	*LOC101121659*
2	126.30–126.35	0.5132	37.06	2.0021	*LOC101117569* *LOC114113454*
2	126.80–126.85	0.6172	68.42	1.5837	*LOC114113042*
2	128.50–128.55	0.4913	37.97	1.8451	*MFSD6, NEMP2*
2	128.65–128.70	0.4222	69.54	2.5183	*INPP1*
2	128.70–128.75	0.3829	50.98	2.0698	*LOC114113048, LOC114113049*
2	128.80–128.85	0.3925	40.06	1.8742	*HIBCH*
2	129.05–129.10	0.3761	36.78	1.7900	*MSTN*
2	129.35–129.40	0.4355	37.59	2.0789	*ANKAR, ORMDL1, OSGEPL1*
2	130.06–130.11 130.16–130.20	0.4441 0.3765	57.92 43.93	1.3992 1.6652	*COL5A2*
2	130.20–130.23	0.4852	47.42	1.6461	*COL3A1*
2	130.28–130.30 130.45–130.50	0.4308	52.78	1.8116	*LOC101120147, LOC114113104*
3	135.48–135.50	0.3735	36.51	1.4174	*ATP2B1, LOC101111701*
9	70.40–70.41	0.3736	71.87	1.8503	*TRNAC-GCA, CSMD3*
14	49.05–49.09	0.4501	57.24	1.8655	*HAUS5, RBM42*

Nonsynonymous mutation screening identified 24 functional variants across seven genes (*NEMP2*, *MFSD6*, *INPP1*, *OSGEPL1*, *COL5A2*, *COL3A1*, and *HAUS5*; Table 3). Haplotype-based tagging yielded 100 tag SNPs on chromosome 2. Combined with the missense variants and consensus genes, we established a panel of 145 molecular markers for EFR lactation performance.

**Table 3.** Nonsynonymous mutation loci in the selected region of East Friesian sheep

Gene	Mutation type	Chr	Position	Ref	Alt
*NEMP2*	Nonsynonymous	2	128520082	G	A
*NEMP2*	Nonsynonymous	2	128521743	A	G
*NEMP2*	Nonsynonymous	2	128524519	G	A
*MFSD6*	Nonsynonymous	2	128540117	A	G
*MFSD6*	Nonsynonymous	2	128540151	C	T
*MFSD6*	Nonsynonymous	2	128540157	A	G
*INPP1*	Nonsynonymous	2	128659848	T	C
*INPP1*	Nonsynonymous	2	128659365	C	T
*OSGEPL1*	Nonsynonymous	2	129376530	A	G
*COL5A2*	Nonsynonymous	2	130193848	G	A
*COL3A1*	Nonsynonymous	2	130214624	T	C
*COL3A1*	Nonsynonymous	2	130226140	G	A
*HAUS5*	Nonsynonymous	14	49066052	G	A
*HAUS5*	Nonsynonymous	14	49066342	G	T
*HAUS5*	Nonsynonymous	14	49067956	A	C
*HAUS5*	Nonsynonymous	14	49068010	A	G
*HAUS5*	Nonsynonymous	14	49069252	C	G
*HAUS5*	Nonsynonymous	14	49069696	T	C
*HAUS5*	Nonsynonymous	14	49070126	G	A
*HAUS5*	Nonsynonymous	14	49070187	G	A
*HAUS5*	Nonsynonymous	14	49070219	C	A
*HAUS5*	Nonsynonymous	14	49070461	C	T
*HAUS5*	Nonsynonymous	14	49070526	A	G
*HAUS5*	Nonsynonymous	14	49073117	A	G

### Lactation-stage transcriptomics identifies critical functional genes

**Figure 3.**
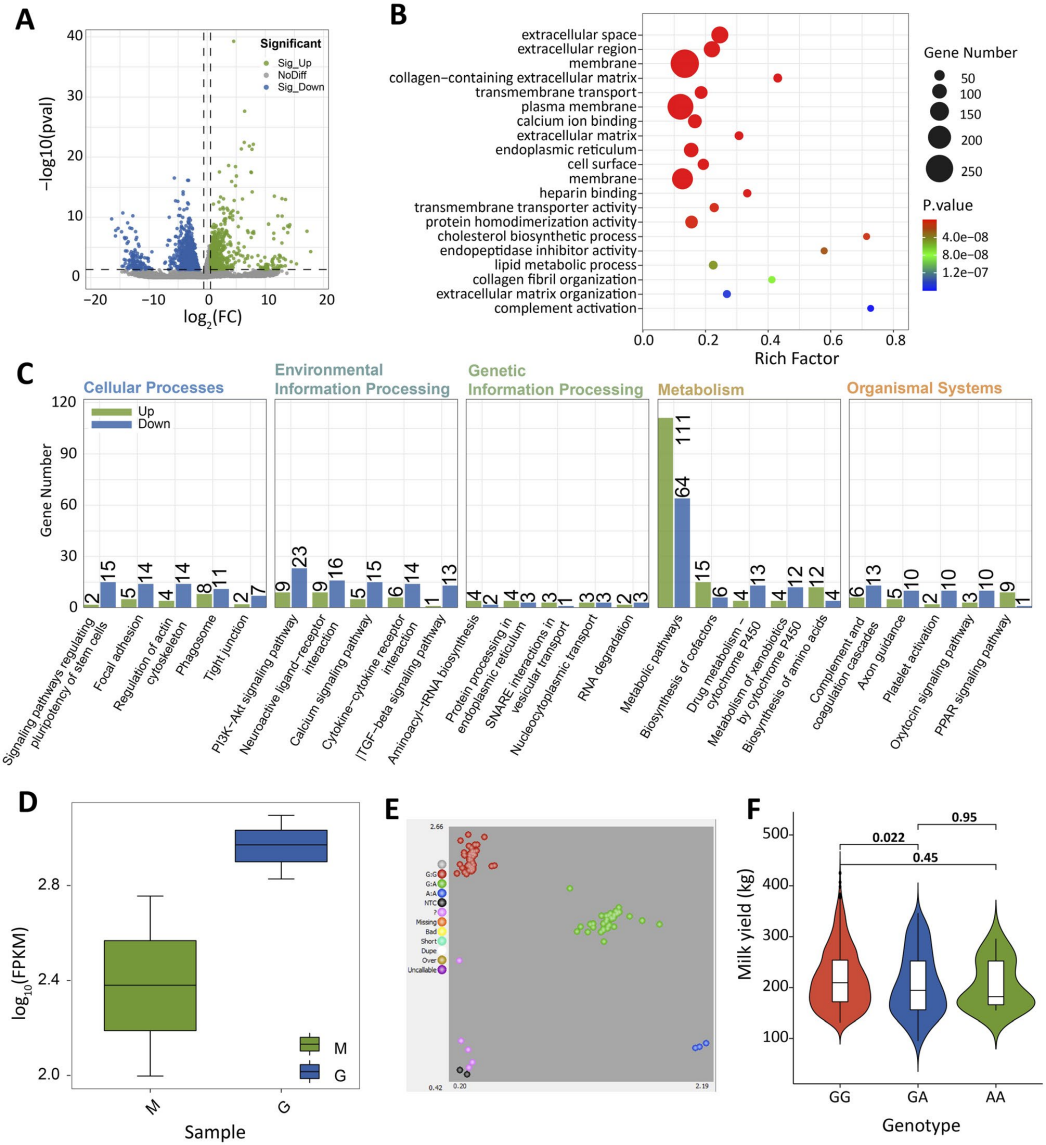
Transcriptome analysis of different lactation stages and validation of *COL3A1* gene in EFR × Hu crossbred dairy sheep. (A) Volcano map of DEGs; (B) GO enrichment analysis; (C) KEGG enrichment pathway; (D) *COL3A1* gene expression; (E) *COL3A1* genotyping map; (F) Association analysis between *COL3A1* gene g.130226140G>A and milk yield.

Mammary tissues from lactation and dry milk stages were screened for DEGs using |log_2_FC| > 1.5 and *P *< 0.05. Overall, 2,178 DEGs were identified, of which 671 were upregulated and 1,507 were downregulated (Fig. 3A; Supplementary Table S6). The upregulated genes included *CSN3* (Li et al., 2024), *LALBA* (Ostrowska et al., 2021), *KIF19* (Chen et al., 2018), *BTN1A1* (Brzáková et al., 2021), *PAEP* (Kolenda et al., 2021), and *FASN* (Poncet et al., 2024). Reportedly, the first four genes are related to milk yield, and the remaining are related to milk composition. The down-regulated genes were *ThymB4X*, *PTN*, *ROR2*, *SLC35A3*, *PLBD1*, *COL3A1*.

Functional enrichment analysis of the 2,178 DEGs identified 878 significant GO terms, comprising 592 biological processes (BPs), 89 cellular components (CCs), and 197 molecular functions (MFs). The most enriched BP terms included transmembrane transport (GO: 0055085) and extracellular matrix organization (GO: 0030198), while CC terms were predominantly associated with collagen-containing extracellular matrix (GO: 0062023) and plasma membrane (GO: 0005886). MF terms showed strong enrichment for calcium ion binding (GO: 0005509) and heparin binding (GO: 0008201), with extracellular space (GO: 0005615) representing the most significantly enriched pathway (Fig. 3B).

KEGG pathway analysis revealed 255 enriched pathways, with metabolic pathways (map01100), PI3K-Akt signaling (map04151), and MAPK signaling (map04010) being shared by both up- and down-regulated DEGs. Upregulated genes were particularly enriched in lipid metabolism pathways (glycerophospholipid metabolism, map00564; PPAR signaling, map03320) and biosynthetic pathways (amino acids, map01230; cofactors, map01240), whereas downregulated genes showed association with stem cell pluripotency regulation (map04550) and calcium signaling (map04020) (Fig. 3C).

These enriched GO terms and KEGG pathways are closely related to mammary gland biology. For instance, extracellular matrix organization and PI3K-Akt/MAPK signaling are essential for mammary gland remodeling and epithelial cell proliferation during lactation, while transmembrane transport and calcium ion binding are directly involved in milk component secretion (Safdar et al., 2024; Tan et al., 2024). Moreover, the enrichment of lipid metabolism (Mu et al., 2022) and amino acid biosynthesis pathways (Wu et al., 2020) reflects the increased demand for milk fat and protein synthesis, highlighting the molecular basis of lactation-associated metabolic adaptation.

Notably, two selection signature genes (*ATP2B1* and *COL3A1*) exhibited differential expression in mammary tissue, with *COL3A1*—containing a nonsynonymous mutation—emerging as a strong candidate for influencing milk production traits through stable inheritance (Fig. 3D).

### Association analysis of *COL3A1* polymorphisms with milk yield

Genotyping of the *COL3A1* g.130226140G>A was performed in 1,019 F2-generation EFR×Hu crossbred dairy sheep using KASP technology (Fig. 3E). Overall of 817 individuals were wild-type (GG), 186 heterozygous (GA), and 16 mutant-allelic (AA) at g.130226140G>A (Supplementary Table S7). Genetic diversity parameters were calculated for g.130226140G>A. The frequencies were 0.802 for wild-type (GG), 0.183 for heterozygous (GA) genotypes, 0.016 for AA genotypes, 0.107 for A genes, 0.893 for G genes, and low polymorphism at the locus (*PIC* < 0.25). To investigate the effect of the g.130226140G>A, different genotypes of *COL3A1* were analyzed with the milk yield (Fig. 3F). Wild-type (GG) showed significantly higher milk yield than heterozygous (GA) (*P *< 0.05), with GA producing 11 kg less per lactation than GG.

## Discussion

With growing consumer demand for high-quality dairy products, sheep milk has gained increasing attention due to its superior nutritional profile. This trend underscores the urgent need to develop improved dairy sheep breeds through targeted genetic selection. Identifying molecular markers associated with lactation traits in EFR sheep using selection signature analysis represents a critical step toward advancing dairy sheep breeding programs, with significant implications for both China’s and the global sheep milk industry. However, current genomic research on EFR sheep remains limited worldwide, and only a small number of candidate genes potentially related to lactation traits have been reported to date—a research gap that constrains the systematic development of superior dairy sheep breeds.

Notably, all studied breeds exhibited white wool (except blackish-gray Gotland sheep), a phenotype that is consistent with the known role of *KITLG* in pigmentation and coat color determination (Amyere et al., 2011; Wu et al., 2021). In addition, *KITLG* has been associated with litter size in goats (Yuan et al., 2019), further supporting its pleiotropic role in economically important traits. Although no direct evidence currently links *KITLG* to lactation, its potential involvement warrants further investigation in dairy sheep. As a ligand for the mammary-expressed *KIT* gene, it maintains epithelial cell differentiation through autocrine signaling (Ulivi et al., 2004). However, its absence in Fst/iHS results and intergenic localization necessitate further validation for dairy trait association. Parallel iHS analysis revealed another candidate gene highly expressed in reproductive and proliferative tissues (testis, placenta, tumors) (McWilliams et al., 2013; Sangiao-Alvarellos et al., 2013; Krsnik et al., 2022), suggesting possible pleiotropic effects on lactation physiology.

Through integrated analysis using three selection signal detection methods, we identified six candidate genes (*TRNAC-GCA*, *HIBCH*, *ATP2B1*, *ANKAR*, *COL5A2*, and *COL3A1*) potentially associated with lactation traits in dairy sheep. Notably, *TRNAC-GCA* has been demonstrated to influence milk composition and yield in buffalo (Buaban et al., 2022), while *HIBCH* shows a strong correlation with milk production in EFR (Li et al., 2022). The *ATP2B1* gene variants are known to affect multiple dairy traits, including fertility, milk yield, and milk components (Rahman et al., 2023). Importantly, we found *COL5A2* and *COL3A1*, two extracellular matrix genes, to be highly relevant to mammary gland function. *COL5A2* is highly expressed in normal mammary tissues (Paul et al., 2022) and forms part of the critical mammary extracellular matrix network (Li et al., 2020). *COL3A1* plays a role in mammary gland development and shows differential expression patterns during lactation cycles (Chen et al., 2020b). QTL mapping revealed that both collagen genes completely overlap with milk protein percentage regions in sheep, with identified missense mutations potentially representing causal variants. Additionally, we detected multiple missense mutations in *HAUS5*, a gene recently implicated in breast cancer prognosis (Huang et al., 2022), though its specific role in ovine mammary function remains to be elucidated. While these findings highlight several promising candidate genes, it is noteworthy that many other genes identified in our selection sweep analyses in EFR sheep have not been previously associated with lactation or fertility traits. This suggests that EFR sheep may possess additional valuable genetic characteristics beyond dairy production, underscoring the need for further functional studies to fully characterize their genetic potential.

As the transcriptome analysis was conducted on F2 (EFR × Hu) crossbred individuals rather than purebred EFR sheep, it is important to note that the F2 generation carries approximately 75% EFR ancestry and exhibits a milk production performance comparable to that of EFR crosses, with an average yield of ≥350 kg per lactation. This ensures that the transcriptome data are representative of high-yielding milk production traits. Overall, 2178 DEGs were identified during lactation and dry milking in dairy sheep. The gene encoding κ-Cn, *CSN3*, is upregulated during lactation, and several reports have confirmed that this gene is an important candidate for influencing milk production traits and that mutations in the gene are also associated with the protein content in milk. Milk production can be improved through breeding selection programs that include polymorphisms in this gene (Jawasreh and Al-Amareen, 2023). The *LALBA* gene encoding whey protein alpha, which is involved in the synthesis of lactose synthase in the mammary gland and promotes milk production and secretion, is also upregulated during lactation and is therefore considered a plausible candidate for milk yield (Čítek et al., 2023). In addition, many genes affecting milk composition and dairy products have been used as candidate genes affecting milk production, such as the *PAEP* gene (p.Tyr38His), which affects milk protein and milk fat rate of goat milk, the *FASN* gene affects milk fat synthesis, and the *SCD* gene affects milk fat content; all are upregulated during lactation (Arnyasi et al., 2009). Pathway enrichment analysis yielded particularly insightful results: upregulated genes showed strong association with the PPAR signaling pathway, known to govern mammary epithelial cell proliferation, whereas downregulated genes were predominantly linked to the PI3K–Akt pathway that regulates mammary gland involution (Xuan et al., 2022). These findings collectively provide mechanistic insights into the molecular networks underlying ovine lactation physiology.

Integrated transcriptomic and selection signal analyses identified two functionally significant genes—*ATP2B1* and *COL3A1*. *ATP2B1*, a predicted target of miR-99a-3p, regulates calcium homeostasis in mammary epithelial cells through the miR-99a-3p/ATP2B1 axis, influencing colostrum calcium levels (Chen et al., 2020a). Notably, *COL3A1* emerged as a multifunctional candidate. It shows differential expression, selection signatures, and missense mutations. As a key extracellular matrix component, it participates in mammary gland remodeling and development, with demonstrated importance in buffalo (Choudhary et al., 2019) and sheep (Chen et al., 2020b) mammary physiology. Mechanistically, *COL3A1* modulates the AKT/mTOR pathway (Kuivaniemi and Tromp, 2019; Wang et al., 2014), crucial for lactation regulation. Notably, the *COL3A1* g.130226140G>A polymorphism showed significant association with milk yield in EFR×Hu sheep, with wild-type individuals producing 11 kg more milk than heterozygous counterparts, suggesting its potential as a molecular marker for dairy traits.

## Conclusion

This study combined selective sweep and transcriptome analyses to identify key genes affecting milk production in dairy sheep, highlighting *COL3A1* polymorphisms as valuable markers for breeding. While our findings provide practical tools for genetic improvement, they are limited to a specific population, and further functional validation across breeds will be needed to confirm their broader applicability.

## Supplementary Material

skaf339_Supplementary_Data

## Data Availability

All relevant data supporting the conclusions of this study are available in full within the manuscript and Supplementary materials. The raw sequencing data generated in this study have been deposited in the NCBI Sequence Read Archive (SRA) under the accession number PRJNA883597.

## Abbreviations

DEGs: Differentially expressed genes
SNP: Single-nucleotide polymorphism
GO: Gene ontology
GWAS: Genome-Wide Association Study
KASP: Kompetitive Allele Specific PCR
KEGG: Kyoto Encyclopedia of Genes and Genomes
NCBI: National Center for Biotechnology Information
QTL: quantitative trait loci
