# Correction to: Selective sweep and mammary transcriptomics identify breed-specific lactation drivers in East Friesian dairy sheep

**DOI:** 10.1093/jas/skag027

**Published:** 2026-03-02

This is a correction to: Danni Li, Xueyang Zhao, Yuhang Xiao, Ran Li, Yu Jiang, Lei Zhang, Yuxuan Song, Selective sweep and mammary transcriptomics identify breed-specific lactation drivers in East Friesian dairy sheep, *Journal of Animal Science*, Volume 103, 2025, skaf339, https://doi.org/10.1093/jas/skaf339

The following change has been made to the paper. The second sentence of the **Abstract** is corrected in the Arabic numbers to read: “Whole-genome resequencing data from 45 EFR sheep were combined with published whole-genome data from 35 additional sheep.” instead of: “Whole-genome resequencing data from 35 EFR sheep were combined with published whole-genome data from 45 additional sheep.”

